# 5-*N*-Carboxyimino-6-*N*-chloroaminopyrimidine-2,4(3*H*)-dione as a hypochlorite-specific oxidation product of uric acid

**DOI:** 10.3164/jcbn.18-6

**Published:** 2018-07-11

**Authors:** Sayaka Iida, Yorihiro Yamamoto, Chisato Susa, Kana Tsukui, Akio Fujisawa

**Affiliations:** 1 School of Bioscience and Biotechnology, Tokyo University of Technology, 1404-1 Katakura-cho, Hachioji, Tokyo 192-0982, Japan

**Keywords:** hypochlorite, uric acid, oxidative stress, nucleophile, allantoin

## Abstract

Although uric acid is known to react with many reactive oxygen species, its specific oxidation products have not been fully characterized. We now report that 5-*N*-carboxyimino-6-*N*-chloroaminopyrimidine-2,4(3*H*)-dione (CCPD) is a hypochlorite (ClO^−^)-specific oxidation product of uric acid. The yield of CCPD was 40–70% regardless of the rate of mixing of ClO^−^ with uric acid. A previously reported product, allantoin (AL), was a minor product. Its yield (0–20%) decreased with decreasing rate of mixing of ClO^−^ with uric acid, indicating that allantoin is less important *in vivo*. Kinetic studies revealed that the formation of CCPD required two molecules of ClO^−^ per uric acid reacted. The identity of CCPD was determined from its molecular formula (C_5_H_3_ClN_4_O_4_) measured by LC/time-of-flight mass spectrometry and a plausible reaction mechanism. This assumption was verified by the fact that all mass fragments (*m/z* −173, −138, −113, and −110) fit with the chemical structure of CCPD and its tautomers. Isolated CCPD was stable at pH 6.0–8.0 at 37°C for at least 6 h. The above results and the fact that uric acid is widely distributed in the human body at relatively high concentrations indicate that CCPD is a good marker of ClO^−^ generation *in vivo*.

## Introduction

Oxidative stress is associated with lipid peroxidation,^(1)^ DNA damage,^(2)^ and protein carbonylation,^(3)^ and thus can cause many diseases such as cancer,^(4)^ diabetes,^(5)^ Alzheimer’s disease,^(6)^ and ischemia reperfusion injury.^(7,8)^ Since oxidative stress is initiated by the formation of reactive oxygen species (ROS), identification of specific ROS *in vivo* is important in pathological studies.

**Fig. 1:**
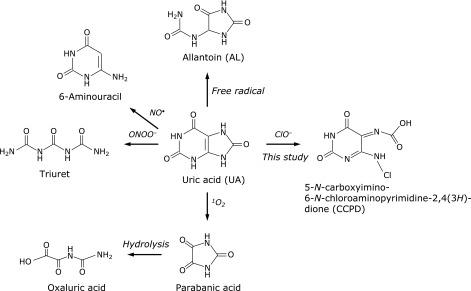
Reported oxidation products of UA induced by reactive oxygen species: AL is produced by free radical-induced oxidation; triuret by ONOO^−^; 6-aminouracil by NO^•^; parabanic and oxaluric acids by singlet oxygen (^1^O_2_); and 5-*N*-carboxyimino-6-*N*-chloroaminopyridine-2,4(3*H*)-dione (CCPD) by ClO^−^ (this study).

For identifying ROS *in vivo*, detection of ROS-specific oxidation products of endogenous antioxidants is a reasonable strategy. Uric acid (UA, Fig. 1) is a suitable substrate for this purpose. Uric acid, which is a terminal metabolite of purine in primates including humans, is widely distributed in body fluid at relatively high concentrations. It reacts with various ROS^(9–11)^ to afford specific products (Fig. 1), e.g., free radical-induced oxidation gives allantoin (AL),^(12)^ ONOO^−^-induced oxidation yields triuret,^(13)^ and nitric oxide (NO^•^) gives 6-aminouracil.^(14)^ Recently, we identified parabanic acid as a singlet oxygen-specific oxidation product of UA and demonstrated its formation on human skin surfaces after sunlight exposure.^(15)^ On the other hand, a hypochlorite (ClO^−^)-specific oxidation product of UA has not yet been characterized.

ClO^−^ oxidizes sulfide to sulfoxide,^(16)^ converts hydrogen peroxide (H_2_O_2_) to singlet oxygen,^(17)^ and chlorinates tyrosine to 3-chlorotyrosine.^(18)^ Myeloperoxidase released from activated neutrophils catalyzes the reaction of Cl^−^ with H_2_O_2_ to form ClO^−^, showing strong microbicidal action against germs including bacteria and Norwalk virus. However, excess ClO^−^ causes oxidative damage to living tissues, especially under acute inflammatory conditions.

In this study, we focused on a ClO^−^-specific oxidation product of UA and identified it as 5-*N*-carboxyimino-6-*N*-chloroaminopyridine-2,4(3*H*)-dione (CCPD, Fig. 1) using time-of-flight mass spectrometry (TOFMS) and a plausible reaction mechanism. The yield of CCPD was 40–70%. Isolated CCPD was stable at pH 6.0–8.0 at 37°C for 6 h. The above results and the fact that UA is widely distributed in the human body at relatively high concentrations indicate that CCPD is a good marker of ClO^−^ generation *in vivo*.

## Materials and Methods

### Chemicals

UA, NaOCl, and other chemicals were purchased from Wako Pure Chemical Industries, Ltd. (Osaka, Japan) and used as received. The concentration of NaOCl was determined as 1.95 M by titration with 0.1 M sodium thiosulfate.

### Reaction of UA and ClO^−^

The reaction of UA and ClO^−^ was conducted at room temperature. UA (220–1,000 µM) was dissolved in 30 ml of 100 mM phosphate buffer solution (pH 7.4) and the solution was stirred by a magnetic stirrer. The NaOCl solution (19.5–195 mM) was introduced into the UA solution (30 ml) at a constant rate (0.25–2.08 µl/min) using a syringe pump (Harvard Apparatus, Holliston, Massachusetts) or added instantaneously to the UA solution. Decay of UA and formation of an unknown product (U1) were monitored by HPLC, LC/TOFMS, and LC/MS/MS, as described below.

### HPLC analysis and isolation

UA, U1, and AL were measured by a reversed-phase HPLC equipped with a UV detector monitoring the absorption at 210 nm. The mobile phase was aqueous ammonium acetate (40 mM) delivered at a rate of 1.0 ml/min. An ODS column (Capcellpak C18, UG80, Shiseido, Tokyo, Japan; 5 µm, 4.6 mm × 250 mm) was used for separation. Retention times for UA, U1, and AL were 7.8, 6.0, and 2.5 min, respectively.

For the isolation of U1, a preparative HPLC system was used. The mobile phase and the separation column were aqueous ammonium acetate (40 mM) delivered at a rate of 3.0 ml/min and an ODS column (Supelcosil SPLC-18, Sigma-Aldrich Japan, Tokyo, Japan; 5 µm, 250 mm × 10.0 mm), respectively. The retention time of U1 was 6.0 min and the elution containing U1 was collected. The U1 fraction was further purified by HPLC as follows. The mobile phase was 15% methanol delivered at 1.0 ml/min. The separation column was a Develosil C30-UG column (Nomura Chemical Co., Ltd., Tokyo, Japan; 5 µm, 250 mm × 4.6 mm). The fractionation was monitored by the absorption at 210 nm. Solvents of U1 fractions were removed under N_2_ gas flow. U1 was then redissolved in water and stored at 4°C.

### LC/TOFMS analysis

To obtain accurate mass-to-charge ratios (*m/z*) of U1, HPLC combined with TOFMS (JMS-T100LC, JEOL, Ltd., Tokyo, Japan) was used. Negative ionization was performed by electrospray ionization (ESI) at an ionization potential of −2,000 V. The optimized applied voltages to the ring lens, outer orifice, inner orifice, and ion guide were −5 V, −10 V, −5 V, and −500 V, respectively, for measurement of the U1 dominant ion. Fragmentation was carried out with an applied voltage to the inner orifice at −50 V. To obtain accurate *m/z* values, trifluoroacetic acid (TFA) was used as an internal calibration standard.

### LC/MS/MS analysis

U1 and AL were quantified using an LC/MS/MS system (LCMS-8040, Shimadzu, Kyoto, Japan). Aqueous formic acid (0.2 ml/min, pH 3.5) was used as the mobile phase with a Develosil C30-UG column (Nomura Chemical Co., Ltd., Tokyo, Japan; 5 µm, 250 mm × 2.0 mm). Negative ionization was performed at −3.2 kV using an electrospray probe. For identification and quantification of each compound, multiple reaction monitoring measurements were obtained. Optimized combinations of product and precursor ions for U1 and AL were determined as −110/−217 and −97/−157, respectively. Chromatographic retention times of U1 and AL were 35 and 4.5 min, respectively.

### Stability of CCPD in solution

The isolated CCPD was dissolved in phosphate buffered solutions adjusted to various pHs (6.0, 7.0, 7.4, and 8.0). Each solution was stored at 37°C or room temperature and the change in the CCPD concentration was determined by HPLC for 6 h or 7 days, respectively.

## Results and Discussion

### Primary product of ClO^−^-induced oxidation of UA

**Fig. 2:**
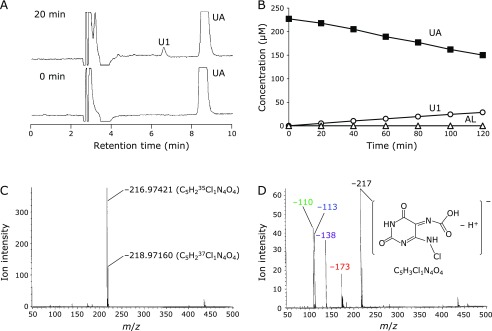
(A) HPLC chromatograms of 100 mM phosphate buffer (pH 7.4) containing UA (230 µM) before (lower panel) and after the continuous addition of NaOCl (27 µM total) for 20 min (upper panel). (B) Time course of changes in concentrations of UA (■), U1 (◯), and AL (△) during the continuous addition of NaOCl (1.35 µM/min) in 100 mM phosphate buffer (pH 7.4) containing UA (230 µM). (C) MS spectrum of U1 measured by LC/TOFMS. Individual *m/z* values were corrected using TFA as an internal standard. (D) Fragmentation pattern of the isolated U1 measured by optimized LC/TOFMS. Fragmentation was performed by collision-induced dissociation with increasing collision energy by changing the inner orifice potential of −10 V to −50 V.

When 100 mM phosphate buffer (pH 7.4) containing UA (230 µM) was mixed with NaOCl continuously (1.35 µM/min) using a syringe pump, an unidentified peak U1 was observed on the HPLC chromatogram of 20 min after the beginning of NaClO introduction (Fig. 2A). The peak increased over time with the concomitant decrease of UA, but no formation of AL was observed (Fig. 2B). The reaction mixture was analyzed by LC/TOFMS with negative ESI and the MS spectrum of U1 is shown in Fig. 2C. The accurate *m/z* value of the dominant anion was determined to be −216.97421 using TFA as an internal standard. Therefore, the chemical formula of U1 was estimated as C_5_H_3_ClN_4_O_4_ and the presence of Cl was indicated by the monoisotopic *m/z* of the ^37^Cl derivative (*m/z* = −218.97160). We next purified U1 using two different reversed-phase HPLC conditions as described in Materials and Methods. LC/TOFMS analysis of isolated U1 gave four fragment ions whose *m/z* values were −172.98242, −137.99124, −112.99384, and −109.99697 (Fig. 2D) and their molecular formulas were estimated as [(C_4_H_3_ClN_4_O_2_, C_4_HN_3_O_3_, C_3_H_2_N_2_O_3_, and C_3_HN_3_O_2_) – H^+^], respectively.

### Kinetic studies

Next, we compared rates of NaOCl introduction (R_i_) and UA decomposition (R_d_) because the R_i_/R_d_ ratio indicates the pseudo-stoichiometric number of the reaction (Table 1). The R_i_/R_d_ values were approximately 2 at low R_i_ conditions (<1.35 µM/min), indicating that one molecule of UA reacted with two molecules of ClO^−^. In other words, two molecules of ClO^−^ are required for the formation of one molecule of CCPD. When R_i_ was greater than 6.50 µM/min, AL was detected as a byproduct and the R_i_/R_d_ values increased to ~2.7, indicating that formation of one molecule of AL requires at least 3 molecules of ClO^−^. This was also the case in instantaneous mixing (Table 1). However, we will not go into details of this since AL is not a ClO^−^-specific major oxidation product of UA.

### Mechanism and the product of UA oxidation by two molecules of ClO^−^

Kinetic studies revealed that U1 (C_5_H_3_ClN_4_O_4_) is produced from the reaction of one molecule of UA (C_5_H_4_N_4_O_3_) with two molecules of ClO^−^.

C_5_H_4_N_4_O_3_ + 2 ClO^−^ = C_5_H_4_Cl_2_N_4_O_5_^2−^ (1)

C_5_H_4_Cl_2_N_4_O_5_^2−^ – C_5_H_3_ClN_4_O_4_ = HO^−^ + Cl^−^ (2)

Therefore, HO^−^ and Cl^−^ can be eliminated from the reaction product.

**Fig. 3:**
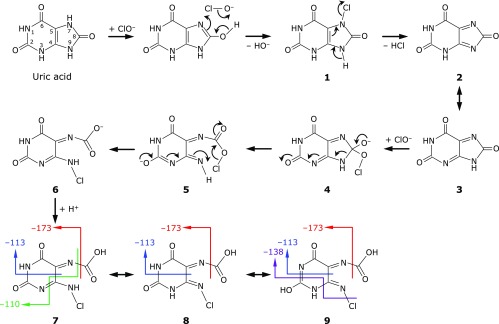
A plausible formation mechanism of CCPD (**7**) in the oxidation of one molecule of UA with two molecules of ClO^−^. Fragmentations of CCPD (**7**) and its tautomers (**8** and **9**) giving ions of *m/z* −173, −138, −113, and −110 are colored in red, purple, blue, and green arrows, respectively.

A proposed reaction scheme is shown in Fig. 3. The lactim (N=C–O–H) of UA and ClO^−^ form a 6-membered ring and release HO^−^ to give the chloramine adduct of UA (**1**), and this adduct releases HCl to produce 1-*H*-purine-2,6,8(3*H*)-trione (**2**). Intermediate **2** is tautomerized to 1-*H*-purine-2,6,8(9*H*)-trione (**3**). Nucleophilic attack on the C8 carbonyl carbon by a second ClO^−^ gives rise to an OCl adduct (**4**). Cleavage at the C8-N9 bond results in the formation of intermediate **5**, which is isomerized to a carboxyl anion (**6**) and then protonated to form 5-*N*-carboxyimino-6-*N*-chloroaminopyrimidine-2,4(3*H*)-dione (CCPD) (**7**). Thus, the release of HO^−^ and HCl and protonation are equal to the elimination of HO^−^ and Cl^−^. As expected, the molecular formula of CCPD is C_5_H_3_ClN_4_O_4_, which is the same as that of U1. CCPD has many tautomers such as **8** and **9**.

To confirm that CCPD is the true ClO^−^-induced oxidation product of UA, matching of 4 fragments [(C_4_H_3_ClN_4_O_2_, C_4_HN_3_O_3_, C_3_H_2_N_2_O_3_, and C_3_HN_3_O_2_) – H^+^] with CCPD was examined. As shown in Fig. 3, all fragments can be found in CCPD and its tautomers (**8** and **9**). Based on the above results, we concluded that CCPD is the ClO^−^-specific oxidation product of UA. It should be noted that ^1^H and ^13^C NMR spectroscopies were not useful to identify this type of compound since there are few protons and the structures of C=O and C=N are oft repeated.

### Stability of CCPD in aqueous solution at various pHs

**Fig. 4:**
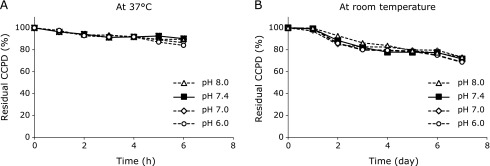
Stability of isolated CCPD in solution at different pHs, 6.0 (◯), 7.0 (◇), 7.4 (■) and 8.0 (△), during storage at 37°C (A) and at room temperature (B).

The effect of pH on the stability of aqueous CCPD solution was examined next. CCPD was very stable at all pHs (6.0–8.0) examined at 37°C for 6 h (Fig. 4A) and relatively stable at room temperature for 7 days (Fig. 4B). These results indicate that CCPD is a good marker of ClO^−^ formation *in vivo*. We plan to apply this probe to plasma samples from patients associated with acute inflammation such as sepsis.

## Conclusions

A ClO^−^-specific oxidation product was produced from two molecules of ClO^−^ and one molecule of UA. It was identified as CCPD by its mass number and plausible reaction scheme and confirmed by mass fragments. Aqueous CCPD was stable at physiological pH. These results suggest that CCPD can be a good indicator of ClO^−^ generation *in vivo*.

## Figures and Tables

**Table 1:** Degradation of UA and the formation of CCPD during continuous or instantaneous addition of NaClO [µM, mean ± SD (*n* = 3)]

Addition of NaClO			[UA]_0_	Time (min)	−Δ [UA]	R_d_ (µM/min)	R_i_/R_d_	[CCPD]	CCPD yield (%)	[AL]	AL yield (%)
Continuous addition	R_i_ (µM/min)	0.16	220	120	9.8 ± 0.2	0.081 ± 0.001	1.99 ± 0.03	5.32 ± 0.17	54.4	ND	0.0
		0.65	230	120	39.0 ± 1.3	0.33 ± 0.02	1.95 ± 0.10	16.8 ± 0.1	45.3	ND	0.0
		1.35	230	120	79.1 ± 1.2	0.66 ± 0.01	2.05 ± 0.03	28.5 ± 0.3	36.1	ND	0.0
		6.5	230	80	197 ± 5.8	2.47 ± 0.07	2.64 ± 0.08	76.4 ± 4.5	38.8	3.7 ± 1.9	1.9
		13.5	1,075	120	587 ± 22	4.89 ± 0.18	2.66 ± 0.10	244 ± 11	41.6	1.7 ± 0.2	0.3
Instantaneous mixing	[NaClO] (µM)	110	250	120	36.3 ± 4.3	—	3.14 ± 0.55^a^	23.4 ± 2.0	66.2	1.4 ± 0.0	3.9
		240	240	120	81.3 ± 1.5	—	2.95 ± 0.04^a^	52.0 ± 0.9	64	11.2 ± 2.1	13.8
		480	250	120	154 ± 1.5	—	3.09 ± 0.02^a^	106 ± 9.3	69	30.5 ± 1.2	19.8

UA, uric acid; CCPD, 5-*N*-carboxyimino-6-*N*-chloroaminopyrimidine-2,4(3*H*)-dione; AL, allantoin; ND, not detected. ^a^value is expressed as [NaClO]/(−Δ[UA]).

## Abbreviations

AL: allantoin
CCPD: 5-*N*-carboxyimino-6-*N*-chloroaminopyrimidine-2,4(3*H*)-dione
ClO^−^: hypochlorite
ESI: electrospray ionization
NO^•^: nitric oxide
R_d_: rate of UA decomposition
R_i_: rate of NaOCl introduction
TFA: trifluoroacetic acid
TOFMS: time-of-flight mass spectrometry
U1: unknown product
UA: uric acid
